# Improved quality of life and pain relief in mature horses with osteoarthritis after oral transmucosal cannabidiol oil administration as part of an analgesic regimen

**DOI:** 10.3389/fvets.2024.1341396

**Published:** 2024-02-06

**Authors:** Claudia Interlandi, Marco Tabbì, Simona Di Pietro, Fabiola D’Angelo, Giovanna L. Costa, Francesca Arfuso, Elisabetta Giudice, Patrizia Licata, Daniele Macrì, Rosalia Crupi, Enrico Gugliandolo

**Affiliations:** ^1^Department of Veterinary Sciences, University of Messina, Messina, Italy; ^2^Freelancer, Varese, Italy; ^3^Zooprophylactic Institute, Palermo, Italy

**Keywords:** horses, cannabidiol, oral transmucosal, osteoarthritis, quality of life, pain, hemp oil

## Abstract

The aim of this study was to evaluate the effect of oral cannabidiol (CBD) administration in addition to a conventional analgesic protocol on the clinical signs of 20 horses with mild joint osteoarthritis. The horses were randomly assigned to either the control group (C group) or the cannabidiol group (CBD group). Both groups were treated with phenylbutazone for 5 days. The CBD group received 0.03 mg/kg cannabidiol in hemp oil orally once daily for 14 days in addition to phenylbutazone treatment. All subjects were monitored for clinical parameters, oxidative status and blood counts. Pain and quality of life were also assessed using the Horse Chronic Pain Scale (HCPS). The CBD group showed a significant reduction in heart rate, respiratory rate, white blood cell count and oxidative stress (malondialdehyde lipid peroxidation). A significant reduction in HCPS scores was seen in both groups. Lower scores were recorded in the CBD group (3 med; range: 2/4) than in the C group (7 med; range: 4/10). The addition of a cannabidiol-based product to an analgesic protocol was well tolerated and showed positive effects on the treated subjects, improving their quality of life and pain relief.

## 1 Introduction

The therapeutic use of cannabidiol (CBD) is becoming increasingly popular, probably due to its perception as a natural treatment among pet owners (1). Several studies have shown that CBD reduces the production of inflammatory cytokines, thus helping to reduce inflammation in various tissues of the body and may have a calming effect on horses (2, 3). Osteoarthritis (OA) is a progressive condition also known as degenerative joint disease. It is a major musculoskeletal disorder, causing reduced mobility and reluctance to move, affecting many species and is particularly prevalent in horses (4–6). These disorders (degenerative arthritis, osteoarthritis of the metacarpal/metatarsophalangeal joint, interphalangeal joints, hoof disorders, vertebral lesions, kissing spines, periarticular osteophytes, synovitis) can lead to real complications with reduced welfare. Horses tend to be reluctant to be handled, unwilling to move and show signs of pain (lameness). These disorders result in economic losses to horse owners. Therapeutic management includes topical treatments, intraarticular injections and systemic analgesics and antiinflammatory therapies (7). Non-steroidal anti-inflammatory drugs (NSAIDs), such as phenylbutazone, are commonly used to treat osteoarthritic pain, but their long-term use is not recommended because the potential side effects of these drugs, such as anorexia, gastric irritation, peptic ulcer, nephrotoxicity, hepatotoxicity and hematological diseases (8–10). As a result, alternative therapies are constantly being sought and new treatments are often proposed. The endocannabinoid system (ECS) is an intercellular communication system responsible for regulating physiological processes in the organism and promoting homeostasis. The medical use of cannabinoids, particularly phytocannabinoids, has been one of the most interesting approaches to pharmacotherapy in recent years (11). The cannabis plant (*Cannabis sativa* L.), a member of the Cannabaceae family, produces numerous compounds, including terpenes, flavonoids, phenolic acids and phytocannabinoids (12, 13). Among the latter, cannabidiolic acid (CBDA) and tetrahydrocannabinolic acid (THCA) are prevalent. During heat extraction they are decarboxylated to cannabidiol (CBD) and 19-tetrahydrocannabinol (THC), which are the most widely used today for their potential medicinal applications (14–17). Cannabidiol (CBD) is the second most important cannabinoid, after ∆9-tetrahydrocannabinol (THC). Although THC and CBD share the same chemical formula, their pharmacological characteristics are not equivalent (18). When given orally, these substances quickly enter the bloodstream where they bind to lipoproteins, albumin and red blood cells. They tend to accumulate in the adipose tissue, liver, lungs, spleen, brain and muscles, from where are continuously released as plasma levels decline. This suggests a long elimination phase of CBD associated with a high volume of distribution to various tissues. Natural derivatives undergo hepatic hydroxylation, decarboxylation and conjugation, and may also be metabolized in extrahepatic tissues such as the small intestine lungs and brain (15–17). The analgesic properties of cannabinoids (CBD; THC) appear to be related to their lipophilic properties, which allow them to easily cross the blood–brain barrier and induce analgesia. However, the mechanism of pain control by cannabinoids is still unclear whether it is due to CB1 and CB2 receptor agonism or to effects caused by interaction with neuromodulators or inhibition of neurotransmitters such as glutamate, dopamine, prostaglandins, acetylcholine, GABA, histamine, noradrenaline and endogenous opioid peptides involved in pain modulation (15–19). The study of oxidative stress plays an essential role in many diseases, and Malondialdehyde (MDA) is one of the best known secondary products of lipid peroxidation, used as an indicator of cell membrane damage. The degree of serum lipid peroxidation was quantified by measuring the concentration of malondialdehyde (MDA). Malondialdehyde reacts with thiobarbituric acid to form a red-pink colored product, which can be measured spectrophotometrically at 535 nm as previously described (19, 20). Cannabinoids have immunomodulatory, antihyperalgesic, antinociceptive, anticancer and anti-inflammatory effects. They also promote tissue regeneration and have neuroprotective properties (3, 21–24). Phytocannabinoids interact with cannabinoid receptors and produce antioxidant and anti-inflammatory effects in the body (11, 24). Compared to oral, intramuscular and intravenous administration, the oral transmucosal route (OTM) is increasingly used for systemic drug delivery because it is easy to perform, fast and painless, and the high blood supply to the oral mucosa avoids hepatic first-pass effect or gastrointestinal degradation (2, 3, 25). The use of cannabidiol (CBD) in various species is gaining popularity as a potential supplement to conventional pharmaceutical treatments for a variety of conditions (2, 26, 27). The aim of the present study was to evaluate the efficacy of CBD 15%, included in a classical pharmacological regimen, in relieving pain in horses with osteoarthritis by monitoring clinical and hematological parameters, oxidative stress and quality of life. The authors hypothesized that low doses of 15% CBD administered into the buccal mucosa could improve the efficacy of an analgesic protocol used to treat OA-related pain in horses, without causing side effects.

## 2 Materials and methods

### 2.1 Animals

The study was approved by the Institutional Ethical Committee for Animal Care of the University of Messina. The animal husbandry and experimental protocols were reviewed and approved according to the standards of the Guide for the Care and Use of Laboratory Animals and Directive 2010/63/EU on animal experimentation (approval no.: 089/2022). All owners were fully informed of the study and gave written consent for their horses to participate. Twenty-four client-owned horses of different breeds, ages, weights and sexes, stabled at the Riding Club “La Palma,” were evaluated and treated for OA-related pain. A baseline screening (T0) was performed, consisting of a physical examination and hematochemical profile to exclude co-morbidities, and the animals were free of internal and external parasites. Horses were eligible for inclusion in the study if they were otherwise healthy, had clinical signs of lameness due to osteoarthritis (metacarpophalangeal or metatarsophalangeal joint) localized to one or more joints diagnosed by “joint locking” and confirmed by imaging techniques, such as radiography or ultrasonography. Radiographic findings and OA localization were noted and recorded by a boarded radiologist. Chronic painful conditions, included OA, were considered mild by the attending veterinarian, using the Horse Chronic Pain Scale (HCPS). Subjects in the groups remained under close veterinary supervision and examinations were conducted in the stables during the day and lasted no more than 30 min in total. The horses were fed a fiber-base diet. Concentrates were not administered to avoid excessive protein intake in resting animals, which could adversely affect their welfare (28, 29). Two weeks before the start of the study, the horses were handled by two of the observers conducting the study, in order to get the animals used to their presence and manipulations. Exclusion criteria included horses suffering from other concomitant diseases, receiving other analgesic therapies or having undergone orthopedic surgery in the 3 weeks prior to the first assessment.

### 2.2 Study design

**Figure 1:**
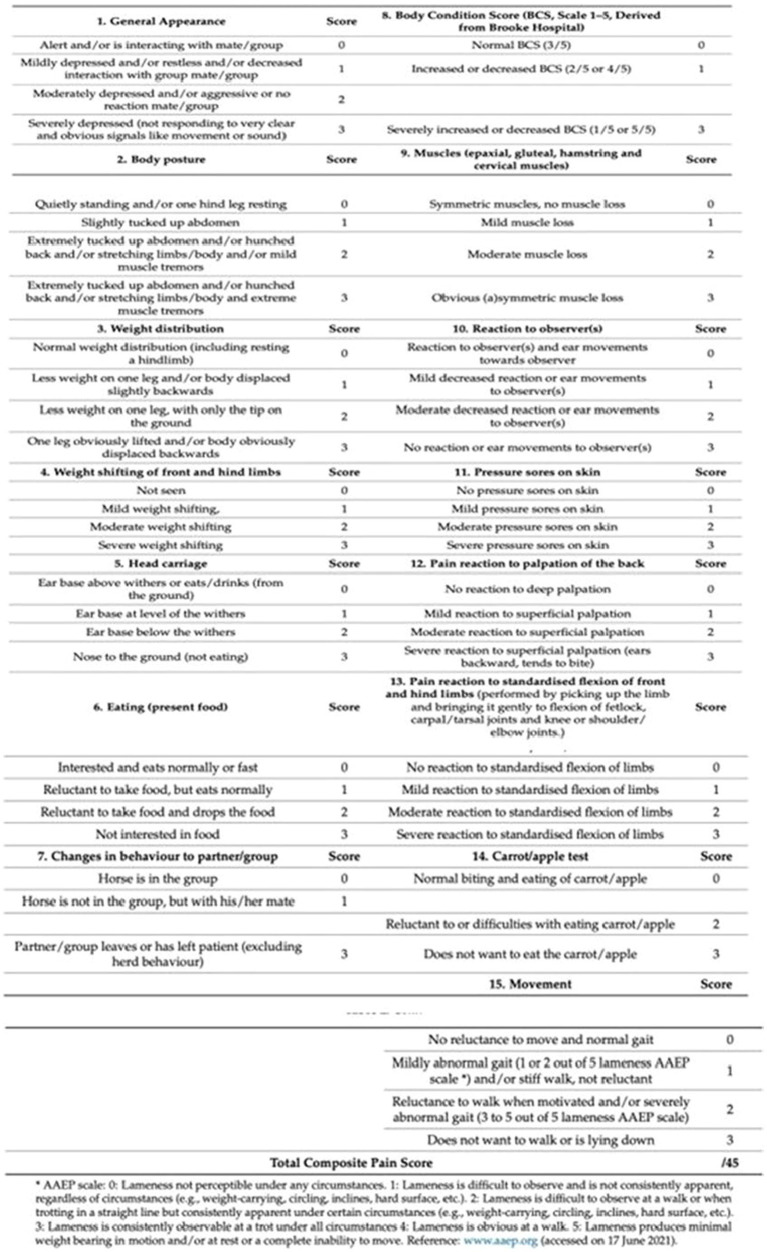
Horse Chronic Pain Scale (HCPS).

Horses were enrolled for 12 months and treated for OA-related pain. All enrolled subjects were randomized using commercial software (Microsoft Office Excel 2013; Microsoft Corp, Redmond, WA, United States) into two groups: a control group (C group) treated with phenylbutazone alone and the group treated with phenylbutazone and CBD 15% (CBD group). A total of 24 horses were included in the study, of which 10 horses affected by osteoarthritis of the metacarpophalangeal joints (right and left), 6 horses affected by osteoarthritis of the metatarsophalangeal joints (right or left or both) and 8 horses affected by osteoarthritis of the metacarpophalangeal joints (right or left). The signalment and clinical conditions are reported in Table 1 (30). The use of other drugs and supplements was discouraged for at least 2 weeks prior to the start of the study. Owners and stable staff were advised that any introduction of new drugs or changes in dosage during the study period would result in/lead to exclusion from the study. Among the subjects who completed the study, there were 8 geldings and 16 females, from three breeds (16 Italian saddle horses, 6 German saddle horses, and 2 Hanoverian horses). The median age of the patients of group C was 19.5 years (range: 15/22, mean: 18.8 ± 2.4) and of group CBD was 20 years (range: 16/22, mean: 20.1 ± 1.7), the median weight of group C was 462 kg (range: 400/567, mean: 467 ± 52.7) and of group CBD was 442 kg (range: 389/540, mean: 453 ± 46.5), the median body condition score, rated on a scale of 1 to 9, of group C was 6 (range: 4/6, mean: 5.4 ± 0.8) and of group CBD was 5(range: 4/6, mean: 5.1 ± 0.8). The two groups did not differ significantly. Table 1 shows data on horses in terms of breed, age, weight, sex and dosage of NSAID and CBD used. Regardless of group, all horses received phenylbutazone (Bute 200 mg/mL, ACME, Italy) endogenously (IV) at a dose of 2.2 mg/kg daily for 5 days (8), with phenylbutazone administered by the stable vet in the evening. In the CBD group, patients also received a cannabinoid product (CBD 15%), emulsified in hemp oil (GREEN CBD Oil 15%, Green Ladybug; Udine; Italy). Cannabidiol oil 15% was administered by the OTM route at a dose of 0.03 mg/kg every 24 h for 2 weeks (2, 31). The oil suspension was used to prepare drops containing 15% CBD corresponding to approximately 7.5 mg CBD per drop. A certificate of analysis for the batch of product used in this study was obtained from the Forensic Toxicology Laboratory, Section of Forensic Medicine, University Hospital, Umberto I General Hospital. The analysis showed a CBD content of approximately 15.5% and THC content of 0.38%, confirming the product’s compliance with quality control measures, including the assessment of contamination by microbes, mycotoxins, pesticides, heavy metals, and solvents. Oral drops (CBD 15% and THC < 0.5%) were commercially available according to the manufacturer’s specifications.^1^ All horses were weighed using a platform scale to determine the appropriate CBD dose based on individual body weight. Body condition scores (BCS) were assigned independently by three observers (32). All subjects were accustomed to being handled and took the drug easily. Access to food was denied 3 h before drug administration and restored 2 h later. Access to water was unrestricted. For 14 consecutive days, the horses in each group were assessed in their natural environment by three observers. Clinical parameters such as heart rate (HR), respiratory rate (RR), arterial blood pressure [systolic arterial pressure (SAP), diastolic arterial pressure (DAP), mean arterial pressure (MAP)] and rectal temperature (T°) were measured. All horses come from the same horse training center, and during all the experimental period were housed individually in their boxes (3.20 × 3.20 m). All animals were fed with hay 11 kg/horse (water was available *ad libitum*). A fiber- base diet is advantageous from the animal’s welfare point of view as it allows horses to express a more natural time balance resulting in more time spent feeding and less energy production leading to a reduction in excitable behavior (28, 29). Data sets (HR, RR, SAP, DAP, MAP, T°) were collected at the same time of day (10.00–12.00) (33) at the following time points: day one (T1; baseline), day two (T2), day three (T3), day four (T4), day five (T5), day six (T6), day seven (T7), day eight (T8), day nine (T9), day ten (T10), day eleven (T11), day twelve (T12), day thirteen (T13) and day fourteen (14). Data collection were performed during spring and autumn seasons, at about 4 horses for month was enrolled. Clinical assessments were performed using a multiparameter monitor (Datex-Ohmeda S/5; Finland), which displayed HR (beats/min) and (SAP, DAP and MAP (mmHg)). Pulse rate (HR) was measured using a pulse oximeter was placed on the upper lip, while non-invasive blood pressure (SAP, DAP and MAP) was measured using an occluding cuff (size 7.2/13 cm) placed on the tail using the oscillometric method. Respiratory rate (RR, breaths/min) was obtained by direct observation of thoracic wall excursions. Body temperature (T, °C) was monitored using a calibrated thermometer inserted into the rectum. To assess the presence of pain and the horses’ quality of life, three trained and independent observers, unaware of the treatment administered, were asked to complete out the Horse Chronic Pain Scale (HCPS). The HCPS is a validated questionnaire consisting of 15 questions assessing various aspects of the horse’s behavior on a numerical rating scale (34, 35). Total pain scores range from zero (no signs of pain) to 45 (maximum pain score). Seven questions assess pain interference with general activity and enjoyment of life. The seven pain interference questions were scored on a numerical scale ranging from 0 (no interference) to 3 (total interference) (Figure 1) (34, 35). Five questions assessed pain severity through body posture, weight distribution, weight shift of fore and hind limbs, pain response to standardized fore and hind limb flexion and movement. In addition, three questions assessed Body Condition Score, muscles, and pressure points on the skin. All questions were scored on a numerical scale from 0 (no response) to 3 (maximum response) (5, 34, 35). Observers were asked to rate the horses using the Horse Chronic Pain Scale scoring system before the start of treatment on day 1 (T1) and every day for 14 days (T14) at the same time (10.00–12.00). If the scale scores increased, the treatment protocol was modified in consultation with the riding veterinarian and the subject was excluded from the study. In addition, observers and stable staff were asked to record the occurrence of any mild, moderate or severe adverse events. After recording all evaluated parameters, the drugs were administered.

**Table 1:** Horses recruited in C (*n* = 12) and CBD (*n* = 12) groups, breed, age, weight, gender, and therapy administered to SID, once daily.

	Group	Breed	Age	Weight	Gender	NSAIDs	CBD
			(Years)	(kg)		Phenylbutazone	(Cannabidiol 15%) SID
1	C	Italian saddle horses	16	400	Females	2.2 mg/kg SID	None
2	C	Italian saddle horses	20	470	Females	2.2 mg/kg SID	None
3	C	Italian saddle horses	16	567	Geldings	2.2 mg/kg SID	None
4	C	Italian saddle horses	21	520	Geldings	2.2 mg/kg SID	None
5	C	German saddle horses	19	430	Females	2.2 mg/kg SID	None
6	C	German saddle horses	22	454	Females	2.2 mg/kg SID	None
7	C	German saddle horses	18	500	Geldings	2.2 mg/kg SID	None
8	C	Italian saddle horses	15	410	Females	2.2 mg/kg SID	None
9	C	Italian saddle horses	20	430	Females	2.2 mg/kg SID	None
10	C	Italian saddle horses	21	490	Females	2.2 mg/kg SID	None
11	C	Italian saddle horses	19	435	Females	2.2 mg/kg SID	None
12	C	Italian saddle horses	15	421	Females	2.2 mg/kg SID	None
13	CBD	Hanoverian horses	20	430	Females	2.2 mg/kg SID	0.03 mgkg^−1^
14	CBD	Italian saddle horses	16	454	Geldings	2.2 mg/kg SID	0.03 mgkg^−1^
15	CBD	Italian saddle horses	21	500	Geldings	2.2 mg/kg SID	0.03 mgkg^−1^
16	CBD	Italian saddle horses	19	410	Females	2.2 mg/kg SID	0.03 mgkg^−1^
17	CBD	German saddle horses	22	430	Females	2.2 mg/kg SID	0.03 mgkg^−1^
18	CBD	German saddle horses	20	490	Geldings	2.2 mg/kg SID	0.03 mgkg^−1^
19	CBD	Italian saddle horses	22	420	Females	2.2 mg/kg SID	0.03 mgkg^−1^
20	CBD	Hanoverian horses	20	400	Females	2.2 mg/kg SID	0.03 mgkg^−1^
21	CBD	Italian saddle horses	21	470	Geldings	2.2 mg/kg SID	0.03 mgkg^−1^
22	CBD	German saddle horses	20	567	Geldings	2.2 mg/kg SID	0.03 mgkg^−1^
23	CBD	Italian saddle horses	15	455	Females	2.2 mg/kg SID	0.03 mgkg^−1^
24	CBD	Italian saddle horses	18	488	Females	2.2 mg/kg SID	0.03 mgkg^−1^

### 2.3 Hematological and inflammatory oxidative stress

Blood samples were collected from all horses immediately after the assessment of the physiological parameters and HCPS score, before the phenylbutazone/CBD or CBD alone administration in the T1, T7 and T14 data points by an operator blinded to the treatment, with the owners’ informed consent. Blood samples (6 mL) were collected from all animals by jugular venipuncture and was divided into two aliquots, one of which was placed in Vacutainer tubes containing ethylenediaminetetraacetic acid (EDTA) (Terumo Corporation, Tokyo, Japan) as anticoagulant, which was used to assess the oxidative stress by assessing lipid peroxidation malondialdehyde (MDA). Another aliquot of blood was placed in a vacuum tube containing EDTA (K3-EDTA, Vacuette^®^, Greiner Bio- One, Kremsmünster, Austria) and used for the assessment of the blood count. After collection, the serum was separated from the blood samples within 15 min and the serum was immediately stored at −80°C. The other aliquot of blood was were immediately refrigerated at 4°C and then the blood count was assessed (within 3–4 h). Hematological and inflammatory oxidative stress tests were performed and evaluated by a qualified veterinarian to ensure that values were within normal physiological limits prior to enrolling horses into the study.

### 2.4 Statistical analysis

An *a priori* sample size calculation was performed to determine the number of horses needed for this study. Sample size was calculated with the G*Power 3.1 Software (Heinrich-Heine-Universitat Dusseldorf, Düsseldorf, Germany). Accepting an effect size (f) of 0.6, a significance level (α) of 0.05, a power (1-β) of 0.80 with Anova Fixed effects, omnibus, one-way test, 12 horses in each group were necessary to recognize a statistically significant a difference. Statistical analysis was performed using SPSS 21.0 (IBM Company, Italy). The assumption of clinical data normality was examined by a Shapiro–Wilk test. The scores were assigned by three observers, unaware of the treatment administered, and Kendall’s concordance coefficient W was calculated. Clinical data (HR, RR, SAP, MAP, and DAP), scores from Horse Chronic Pain Scale and MDA level were reported as median and range. Differences along the timeline were performed using Wilcoxon’s Test. Hematological parameter are reported as mean ± standard error of the mean (SEM) of every observation. One-way ANOVA was used for analysis, followed by the Bonferroni test for multiple comparisons. A *p* < 0.05 was considered significant.

## 3 Results

The total number of subjects involved in the research study was 24, with an effective power of 0.80. Data were not normally distributed. The level of interobserver agreement was high (*W* = 1). No adverse reactions were observed during or after CBD administration. Attention was paid to signs such as sneezing, head shaking, licking, nausea salivation and signs of sedation, lethargy, ataxia, incoordination, increased or decreased appetite, hyperesthesia, urinary incontinence, diarrhea, mydriasis, hypothermia, blepharospasm, photophobia or nystagmus. Regarding the clinical parameters collected, a significant decrease in heart rate (HR) was observed in the CBD group from day 9 (T9) to the end of the observation period (T14) compared to the baseline values on day 1 (T1, *p* = 0.000), while the heart rate in group C remained constant throughout the observation period. Comparing the two groups shows that in the CBD group the HR is significantly lower than in the C group on T12, T13 and T14 (*p* < 0.05) (Table 2). Respiratory rate (RR) in group C showed no significant change over the two-week period, whereas in the CBD group, RR decreased significantly on several monitoring days (T5, T7, T9, T10, T11, T12, T13, T14, T15) compared to T1 (*p* = 0.000). Comparison of the C Group with the CBD Group showed significantly different RR values, with higher RR values in group C than the CBD Group at T11, T 12, T 13, and T14 (*p* < 0.05). Non-invasive blood pressure: Systolic (SAP), Diastolic (DAP), and Mean (MAP) blood pressure did not differ significantly between the two groups at any time (Table 2). No significant alterations of body temperature (T, °C) were found during the monitoring period. Although some statistically significant results were observed, in all subjects the monitored clinical parameters were within physiological ranges. Total pain scores from the 15 Horse Chronic Pain Scale (HCPS) questions for subjects in each group are shown in Table 3. The reported results show that there was no difference in scores between the two groups at baseline (T1), while there was a significant reduction in HCPS scores from T3 to T14 in both groups (*p* = 0.000). The comparison between the two groups shows a significant reduction at various times in the CBD group compared to the C group T9, T10, T11, T12, T13, T14 (*p* = 0.000). The WBC count remained within the reference ranges, but there was a slight increase in the WBC count in the C group and a slight decrease in the CBD group.

**Table 2:** Clinical parameters.

		Monitoring day													
		T1	T2	T3	T4	T5	T6	T7	T8	T9	T10	T11	T12	T13	T14
Variables															
HR (beats minute)	Group C	44	43	44	40	40	42	43	40	40	40	42	43	40	42
		32/48	36/44	32/44	36/48	36/44	36/44	36/44	32/44	36/44	32/44	36/44	36/44	36/44	36/44
	Group CBD	44	40	44	44	44	40	40	40	40*	40*	38*	36*°	36*°	34*°
		40/48	40/52	36/52	40/52	36/48	36/44	36/44	36/44	36/44	36/40	36/40	32/40	32/40	32/36
RR (breaths minute)	Group C	18	19	18	16	16	18	16	20	16	16	20	16	18	16
		16/24	16/24	16/24	12/24	12/24	12/24	10/24	8/20	8/20	8/16	12/20	12/24	12/24	12/24
	Group CBD	18	18	18	16	16*	16	16*	20	16*	16*	14*°	13*°	13*°	14*°
		16/20	16/20	16/24	12/24	12/20	12/20	10/16	8/20	8/20	8/16	8/18	8/16	8/16	12/16
SAP (mmHg)	Group C	99	101	100	101	101	99	101	100	99.5	100	100	102	103	100
		96/122	94/120	96/118	96/118	94/118	95/120	96/118	95/116	94/118	95/116	92/116	96/114	92/116	92/110
	Group CBD	101	101	99	99	99	97.5	97	98	96.5	97.5	98	99.5	98	100
		95/120	94/120	94/118	95/118	92/118	94/120	96/118	96/116	94/118	96/116	95/116	96/110	96/112	94/110
MAP (mmHg)	Group C	70	69.5	71	71.5	70.5	70	70	71.5	71	70	72.5	71.5	70	70.5
		64/77	63/77	65/78	66/77	60/78	64/78	66/77	64/76	65/78	63/79	64/79	64/78	62/78	66/76
	Group CBD	70	69.5	71	68.5	70.5	70	69.5	71.5	68	70	69.5	71.5	70	70.5
		64/78	63/77	65/78	66/77	60/78	64/78	64/77	64/76	63/78	63/79	64/76	64/78	62/78	66/76
DAP (mmHg)	Group C	52.5	52.5	51.5	51	52	53.5	53.5	52.5	53.5	49.5	51.5	52.5	51.5	51
		44/57	45/58	43/57	45/57	44/57	43/59	42/56	42/57	44/57	43/58	45/58	47/58	44/56	43/56
	Group CBD	53.5	52	52.5	53	51.5	51.5	53	52	52	51	53.5	53	52.5	53
		44/58	43/56	44/58	46/57	44/59	42/62	49/60	44/59	48/60	43/61	48/58	46/59	49/57	49/58

HR, heart rate; RR, respiratory rate; SAP, MAP, and DAP, non-invasive blood pressure; monitored for 14 days were expressed with median and range. *Significant difference in time line compared to day I (T1) values; °Significant difference between groups (*p* < 0.05).

**Table 3:** Horse Chronic Pain Scale (HCPS).

		Monitoring day													
		T1	T2	T3	T4	T5	T6	T7	T8	T9	T10	T11	T12	T13	T14
Pain score	Group C	18	17	15*	15*	15*	13*	12*	10*	9*	7.5*	7*	7*	7*	7*
		12/19	12/18	7/17	7/17	5/17	5/15	3/15	3/12	3/12	3/10	3/9	3/9	3/9	4/10
	Group CBD	17	16	15*	15*	14*	13*	12*	9*	5*°	4*°	4*°	4*°	3*°	3*°
		12/18	12/17	7/17	7/ 17	5/15	4/15	4/13	3/12	3/9	3/8	3/6	3/6	2/4	2/4

Total Composite Pain Score of all horses monitored were expressed with median and range. *Significant difference within the group at time points compared to baseline (T1) °Significant difference between groups, *p* < 0.05.

## 4 Discussion

**Figure 2:**
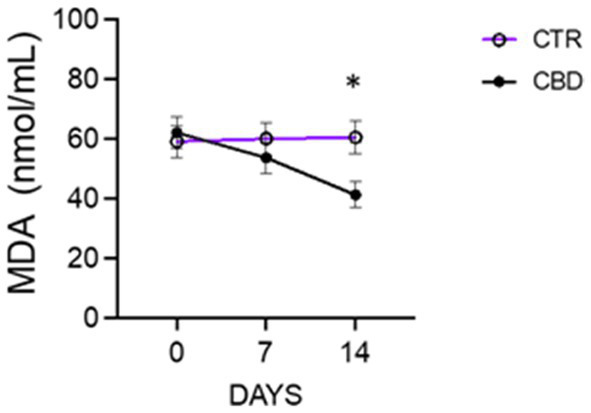
Serum MDA levels in group C and in group CBD; data are showed as mean ± SEM of *n* = 6, *p < 0.05 vs. T1.

Limited information is available on the use of cannabinoid-rich hemp products for pain management in horses with osteoarthritis. Classical therapy involves the use of anti-inflammatory drugs, of which phenylbutazone is one of the most commonly used. The short-term effects of phenylbutazone are well known. However, long-term use of the anti-inflammatory drug on a continuous basis can lead to problems in many cases (8). The first objective of this study was to determine whether the addition of 15% CBD to a basic analgesic protocol would lead to a further improvement in the clinical condition of horses with osteoarthritis. In this study, treatment with 15% CBD in hemp oil in combination with phenylbutazone for 14 days in the CBD group showed a significant decrease in heart rate (HR) and respiratory rate (RR), stable blood pressure, reduced pain scores and serum levels of MDA (a marker of lipid peroxidation) and showed positive results, leading to an improvement in the clinical condition of the patients. Parameters such as heart rate and respiratory rate can be affected by pain and are easy to measure and quantify; however, these parameters alone are not specific to the presence and severity of pain, as they can be influenced by other factors. Several studies have often failed to establish a direct relationship between changes in physiological parameters and the presence or severity of pain. Therefore, these parameters are evaluated in conjunction with other methods to confirm their validity (16, 17, 36–38). In this study, we observed that certain markers of inflammation and oxidative stress changed compared to the treated group. Specifically, as shown in Table 4, we observed that the treatment with CBD for 14 days reduced the number of WBCs, which could be correlated with a reduction in the ongoing inflammatory process. In addition, the data show a significant reduction of the MDA level in the CBD group, probably due to the reduction of inflammation and oxidative stress, an effect that was not observed in the control group (Figure 2). In veterinary medicine, there are few reliable sources on the clinical use of cannabinoids. Although some scientific articles have discussed the potential therapeutic use of CBD in veterinary medicine, few studies have specifically investigated the effects of hemp oil and CBD on chronic orthopedic diseases in horses (39). CB2 analgesia is interesting because it is associated with a peripheral mechanism with no effect on the CNS, in addition to the lack of rapid tolerance compared to that found in CB1 receptors (40). Among the advantages of cannabinoids in horses, CB2 receptors appear to have immunomodulatory properties in that by inhibiting the synthesis/ or release of proinflammatory molecules they modify the inflammatory and infiltrative response in chronic or progressive degenerative joint conditions. Furthermore, their agonists provide an improvement in the treatment of equine veterinary patients with osteoarticular diseases by improving the level of protection against the degenerative process and reducing the main symptomatology, which leads to slowing the progression and outcome of the degenerative condition (40, 41). The authors found that cannabinoid receptors are also distributed in sensory neurons, satellite glial cells (SGCs), macrophages, dorsal root ganglion (DRG) interneurons and synovial elements of the metacarpophalangeal joint in horses. There is a close functional relationship between sensory neurons and the DRG in the peripheral processing of nociceptive input. The results of various studies have encouraged the development of new studies supporting the use of molecules acting on these receptors to reduce joint inflammation in horses. This research has provided an anatomical basis for further studies to explore the therapeutic use of non-psychotropic cannabinoids for pain management in horses (2, 42–44). Research in humans has shown that CBD reduces stress-related signals that can lead to chronic inflammation and pain responses (45). Similar results could be obtained in horses, which are also sensitive to stress as well. Studies in mice, dogs, and horses have shown that CBD reduces the production of inflammatory cytokines such as tumor necrosis factor alpha (TNFα), which may help to reduce inflammation in various tissues and promote a calming effect and reduced anxiety in horses (24, 46, 47). The OTM route has shown good absorption and efficacy of CBD 15% and therefore represents an interesting alternative to other routes of administration as it is a non-invasive and painless technique that allows the use of standardized concentrations and doses, requires minimal constriction and does not cause discomfort to the patient (3, 25, 48, 49). Previous studies have primarily focused on determining appropriate levels of dietary CBD oil supplementation in companion animals, such as dogs and cats, while limited information is available for mature horses. It is clear that small amounts of CBD oil may pass more slowly from the esophagus to the stomach, prolonging its absorption. Conversely, when CBD is administered directly into the mouth, digestion and absorption may be faster. In a study of purebred horses undergoing training, a single oral dose of CBD suspended in sesame oil was administered at 0.5, 1, or 2 mg/kg to evaluate its potential anti-inflammatory effects. Horses tolerated CBD well and no significant behavioral or gastrointestinal abnormalities were observed (2, 31). CBD in an oily suspension formulated for oral and oral transmucosal route administration is currently preferred and seems to be the optimal administration method for efficient absorption (2, 5). In previous research in horses, supplementation with 100 mg CBD over a 6-week period resulted in reduced reactivity (50). Another study administered a single dose of 50, 100, and 250 mg CBD in a pelleted supplement and observed an increase in blood urea nitrogen (BUN), suggesting potential renal stress (51). Given the sensitivity of CBD to temperature and light, the supplements were stored in the dark at a temperature of 4°C until administration (52). Pharmacokinetic studies have shown that CBD has a wide distribution volume and can be found in many tissues and organs (18). Recent studies have shown that CB1 cannabinoid receptors are more widely distributed in the central nervous system, whereas CB2 receptors are mainly located in the periphery. This difference in distribution implies that the pharmacological profile of CB2 receptor agonists is different, with the advantage that CB2 receptors have significant antinociceptive activity with fewer psychoactive side effects, and the anatomical and structural differences between the two receptors may lead to a more appropriate therapeutic target for equine veterinary patients. Both CB1 and CB2 receptors have been found on the cell membrane of intimal synoviocytes and small blood vessels in the subintima of synovial tissue. Some authors have reported changes in the endocannabinoid system (ECS) depending on the degree of synovial inflammation, confirming that both cannabinoid receptors increase with synovitis. However, it has also been observed that with increasing severity of macroscopic joint damage, CB1 expression of synovial cells tends to decrease, but the exact mechanism remains unknown (41). These characteristics would explain CBD’s effect on osteoarthritic pain. Interest in cannabis derivatives in companion animals is growing due to their potential benefits, such as alleviating behavioral problems (53), treating dermatological disorders (54) and managing seizure disorders, thereby improving the quality of life in dogs (55). The results of the present study show that CBD is well tolerated by horses at the doses used. In addition, the degree of CBD absorption has been shown to be influenced by the formulation used, in our study hemp oil was used as the vehicle. Although sesame oil has shown improved absorption in some species, the efficacy of alternative vehicles has not yet been tested in horses. Therefore, the possibility of improved absorption with a different choice of vehicle cannot be excluded (31). The elimination half-life of CBD in horses is slightly prolonged compared with other species, but the primary metabolites are the same as those reported in humans and dogs (31). However, another study reported mild hypocalcemia and increased liver enzymes in all horses receiving supplementation at 0.5 mg/kg or 1.5 mg/kg for 6-week (2, 56). No significant behavioral or gastrointestinal abnormalities were observed in thoroughbred horses in training that received a single oral dose of CBD suspended in sesame oil at 0.5, 1, or 2 mg/kg to evaluate its potential anti-inflammatory effects (2, 31). In humans, food has been shown to influence the extent of CBD absorption (57). As CBD is a highly lipophilic product with low aqueous solubility, administration with food increases its bioavailability, and reduce the daily fluctuation in blood levels of the drug. In the present study, food access was denied 3 h before and 2 h after CBD administration. As horses are less tolerant to high levels of fat in their diet than humans, the influence of food on CBD absorption may be a minor factor. In addition, other authors speculate that even if oral bioavailability was extremely low, as in other species, it is likely that CBD clearance in horses exceeds the hepatic circulation (31, 58). Cannabidiol is a compound that has caught the attention of veterinarians. Although its full pharmaceutical and nutraceutical potential has yet to be realized, the significant presence of this compound in food-grade hemp varieties highlights the need to further investigate its chemical and biological properties and fully exploit its benefits. In a study by Leise et al. (2) horses were given cannabidiol supplementation at 0.12 and 0.13 mg/kg. No substances classified as drugs of abuse, including CBD, 7-arboxy-cannabidiol, 7-Nor-7-carboxy-cannabidiol, and tetrahydrocannabinol (THC), were detected in the blood samples taken at day 0 and 28 days thereafter. This result is significant considering that many horse shows prohibit the use of CBD and cannabidiol. Therefore, the use of CBD at these doses does not pose a risk of disqualification. Several factors influence the welfare of horses, such as appropriate flooring (e.g., sand or grass) and continuous hoof trimming (not more than 6 weeks), access to pasture, sufficient time in the paddock, etc. The horses are always housed in the same boxes (one for the day and one for the night), separated so that they can see each other, to create a stable group of subjects and encourage the formation of a single herd. In addition, the horses are taken out to pasture for a few hours each day to maintain their social bonds (59–61). Furthermore, certain equestrian activities have been associated with a higher risk of limb problems. In addition, certain equestrian activities, such as show jumping, riding and combinations of different disciplines, are associated with a higher risk of limb problems than dressage. In our study, all horses were kept under the same stable conditions and we ensured that the above factors were taken into account. The current study was conducted on horses with mild chronic pain conditions requiring analgesic treatment. This also reflects the management and care vision of the nursing home where the study was conducted, as it supports the principle that horses should be able to live and thrive in daily care without excessive analgesic treatment, but that the use of natural products can improve the quality of life of the treated subjects. A major advantage of using CBD 15% in horses is its availability for sale without restrictions. The broadcast through new social technology, of an innovative natural-based therapy, in particular CBD, could favor its application by a greater number of practitioners and veterinary students (62, 63). This study has some limitations, including the small number of animals and the lack of analysis of CBD plasma concentration during the observation period due to technical constraints. Furthermore, the measurement of serum biochemical parameters (liver transaminases) and urine examination should be evaluated, in addition to considering other biomarkers of inflammation such as C-reactive protein. To identify the analgesia-nociception balance, evaluation of the PTA index would provide reliable data on the true interpretation of heart rate variability (16, 17). In addition, potential sex differences in response to cannabinoids should be investigated. Further investigation would allow the reduction of NSAID doses in the treatment of osteoarthritis to be studied. The effect of habituation cannot be excluded as the horses were closely monitored and the measurements were taken once a day. However, as both groups were subjected to the same monitoring protocol, habituation would have occurred similarly and can therefore be considered negligible. Therefore, the results should be interpreted with caution, and further research is needed to obtain reliable data. Based on the data collected in this study, the addition of 15% CBD to a routine NSAID regimen in horses resulted in a reduction in heart and respiratory rate, WBC count, MDA levels and pain scale scores; all of which could be correlated with a reduction in the ongoing inflammatory process and an improvement in the quality of life of the treated subjects.

**Table 4:** Hematological parameter in group C and in group CBD.

		WBC	RBC	HGB	HCT	MCV	MCH	MCHC	PLT
		(×10^3^/μL)	(×10^6^/μL)	(g/dL)	(%)	(fL)	(pg)	(%)	(×10^3^/μL)
Day	C Group	8.3 ± 1.430	7.65 ± 1.204	12.1 ± 0.997	38.3 ± 4.289	50.8 ± 2.563	16.6 ± 0.825	32.3 ± 3.024	150 ± 32.07
1	CBD Group	8.2 ± 0.364	7.58 ± 0.89	12.1 ± 0.443	38.5 ± 1.20	52.3 ± 1.05	16.5 ± 0.235	31.5 ± 0.167	161 ± 16.3
Day	C Group	8.4 ± 1.511	7.52 ± 1.356	13.0 ± 1.417	39.3 ± 3.730	50.8 ± 2.563	16.7 ± 0.775	32.8 ± 2.878	152 ± 31.97
14	CBD Group	7.2 ± 0.141*	7.21 ± 0.636	11.4 ± 0.526*	34.8 ± 0.335*	51.0 ± 0.966	16 ± 0.284	31.2 ± 0.09	166 ± 5.74

The samples were taken on the first day (T1) and on the last day (14) of the monitoring period. Data are showed as mean ±SEM, *p < 0.05 vs Day 1.

## 5 Conclusion

The aim of the present study was to encourage further research into the multiple effects of CBD, including its analgesic and anti-inflammatory properties. According to our results, oral transmucosal administration of 15% CBD oil (0.03 mg kg^−1^ every 24 h) in addition to intravenous phenylbutazone provided improved pain management and a more satisfactory quality of life than single NSAID treatment in horses with OA. However, further research is needed to fully understand the pharmacodynamics and pharmacokinetics of cannabinoids, to establish a dosage range and to determine potential applications in this species. CBD products could play an important role in alternative or complementary medicine, either as an adjunct to conventional therapeutic approaches or as a stand-alone replacement treatment.

## Data availability statement

The raw data supporting the conclusions of this article will be made available by the authors, without undue reservation.

## Ethics statement

The animal studies were approved by the Institutional Ethical Committee for Animal Care of the University of Messina. The studies were conducted in accordance with the local legislation and institutional requirements. Written informed consent was obtained from the owners for the participation of their animals in this study.

## Author contributions

CI: Conceptualization, Investigation, Writing – original draft, Writing – review & editing. MT: Investigation, Writing – original draft. SP: Data curation, Investigation, Writing – original draft. FD’A: Data curation, Writing – original draft. GC: Data curation, Writing – review & editing. FA: Data curation, Writing – review & editing. EGi: Investigation, Writing – original draft. PL: Writing – review & editing. DM: Investigation, Writing – review & editing. RC: Investigation, Writing – review & editing. EGu: Conceptualization, Writing – review & editing.

## Footnotes

^1^www.greenladybug.it
